# Efficacy of topical application of taurolidine 2% in promoting second intention healing of poststernotomy deep incisional wound infection

**DOI:** 10.1186/s44215-025-00237-y

**Published:** 2025-11-27

**Authors:** Roberto Cemin, Michela Coronet, Andrea Azzolini, Cinzia Viola, Carmen Ladurner, Andrea Comunello, Benito Baldauf

**Affiliations:** 1 Department of Cardiology, San Maurizio Regional Hospital of Bolzano, Bolzano, 39100 Italy; 2 Medical Faculty Christian-Albrechts-Universität, Kiel, Germany; 3 Life Sciences, University of Applied Sciences, An der Karlstadt 8, Bremerhaven, 27568 Germany

**Keywords:** Cardiothoracic surgery, Sternotomy, Wound infection, Taurolidine, Deep incisional, Management

## Abstract

**Background:**

Surgical site infections following median sternotomy are common and can lead to extended hospital stays, prolonged courses of intravenous antibiotics, increased healthcare costs, and, in many cases, the need for repeated debridement or additional surgical interventions. Recognizing this significant unmet clinical need, we adopted an alternative approach in the present case report to address and resolve the problem.

**Case presentation:**

We herein report a case of a 52-year-old female patient, that developed a deep incisional infection with localized dehiscence after a median sternotomy performed for left atrial myxoma removal. Klebsiella aerogenes was cultured from the wound swabs and, despite prolonged intravenous antibiotic therapy and regular debridement we were unable to obtain culture negativity or wound healing over the course of several weeks. A commercially available Taurolidine 2% solution was employed every other day to irrigate the wound, which led to culture negativity within five days. Complete reepithelization was reached after a total of five weeks.

**Conclusion:**

Reports of various taurolidine solutions promoting healing in complex wound lesions are scarcely available. In our case it proved to be an effective therapeutic option, preventing the need for additional surgical intervention and facilitating healing by secondary intention.

## Introduction

Median sternotomy is still the most common access for cardiac surgery. Deep surgical wound infection (DSWI) and mediastinitis after median sternotomy remain significant clinical problems in terms of mortality, morbidity, and healthcare-associated costs [1]. Despite recent advances in medical management and consensus papers, their incidence ranges from 1% to 6%, and the associated mortality ranges from 20% to 50% [2]. The choice of sternal closure technique plays a crucial role in the prevention of DSWI and mediastinitis and should be tailored to the patient’s characteristics. Early aggressive surgical debridement, vacuum-assisted closure (VAC, i.e.; negative pressure therapy) therapy, muscle flap and newer technologies are revolutionizing the paradigm of treatment of DSWI [3]. We herein report a case of chronic wound dehiscence after sternotomy, which resolved after taurolidine 2% irrigation.

## Case presentation

A 52-year-old lady was transferred to our cardiology ward after open heart surgery for a left atrium myxoma. She had undergone medial sternotomy. Her prior medical history was uneventful and she had no comorbidities (BMI 30.5).

**Fig. 1:**
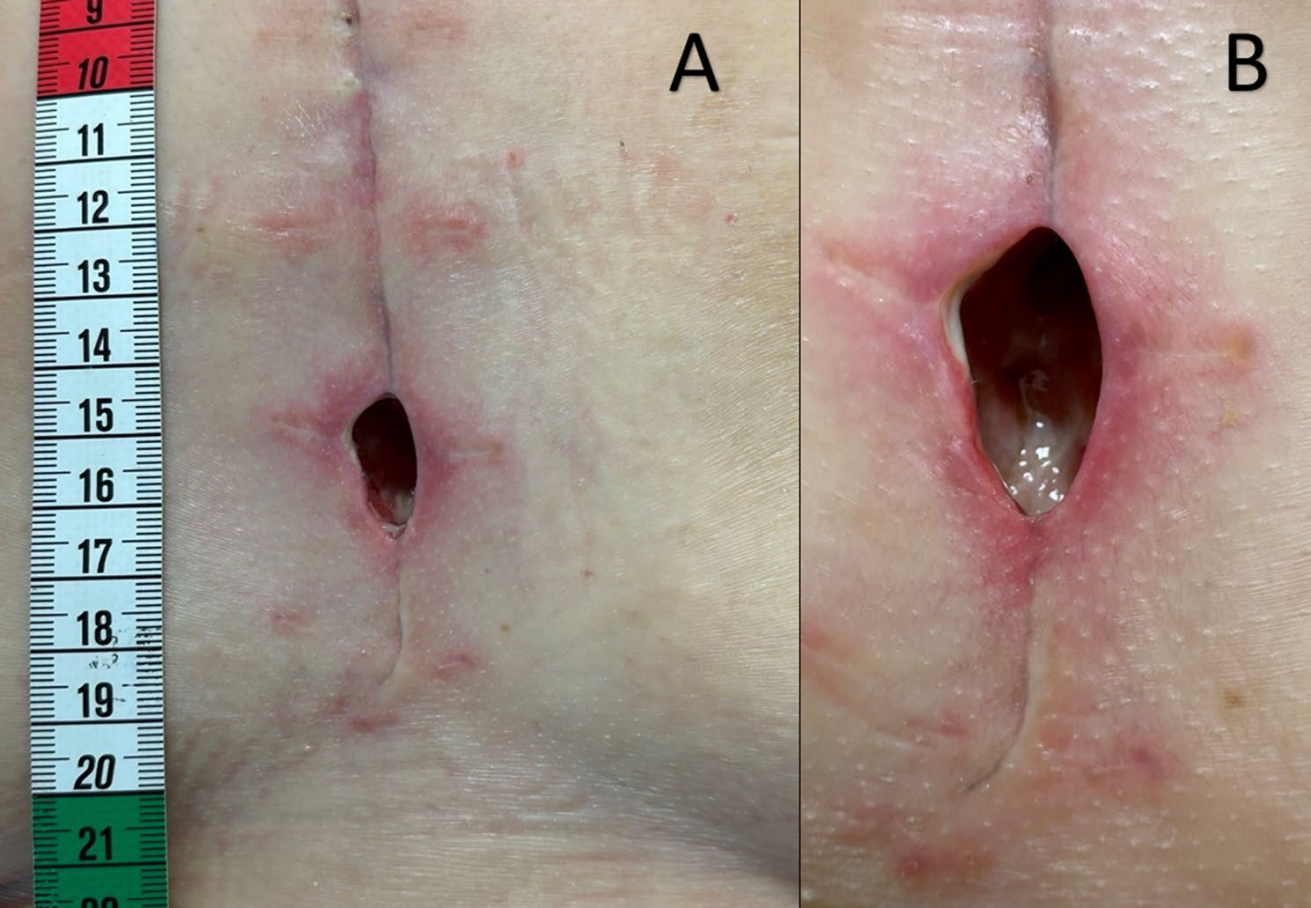
**A** Initial presentation of the sternal wound dehiscence on postoperative day 14, measuring 2 cm in length, 2.5 cm in depth, and 1 cm in width. A wound swab cultured *Klebsiella aerogenes*. **B** Worsening of the wound despite prolonged intravenous antibiotic therapy and repeated antiseptic treatment, with persistent positive cultures and progressive tissue breakdown. Taken on the first day of taurolidine 2% solution treatment, prior to the treatment

On the 14th postoperative day, the sternal wound appeared dehiscent. A chest CT scan revealed an edema/inflammation of the subdermal tissue but excluded mediastinitis. The dehiscence measured 2 cm in length with a depth of 2.5 cm and 1 cm width (Fig. 1a). A swab taken from the dehiscence cultured Klebsiella aerogenes, which resulted producer of inducible cephalosporinase. Despite no fever the patient presented inflammatory markers consistent with bacterial infection. According to the susceptibility screening an antibiotic therapy with 4 g/day of Cefepime (MIC ≤ 0.25) was started.

The heart team consented on surgical wound closure after swab negativity. However, despite prolonged systemic antibiotic therapy and repeated antiseptic treatment, we were unable to sanitize locally. Despite rigorous wound toilet the situs worsened (Fig. 1b).

**Fig. 2:**
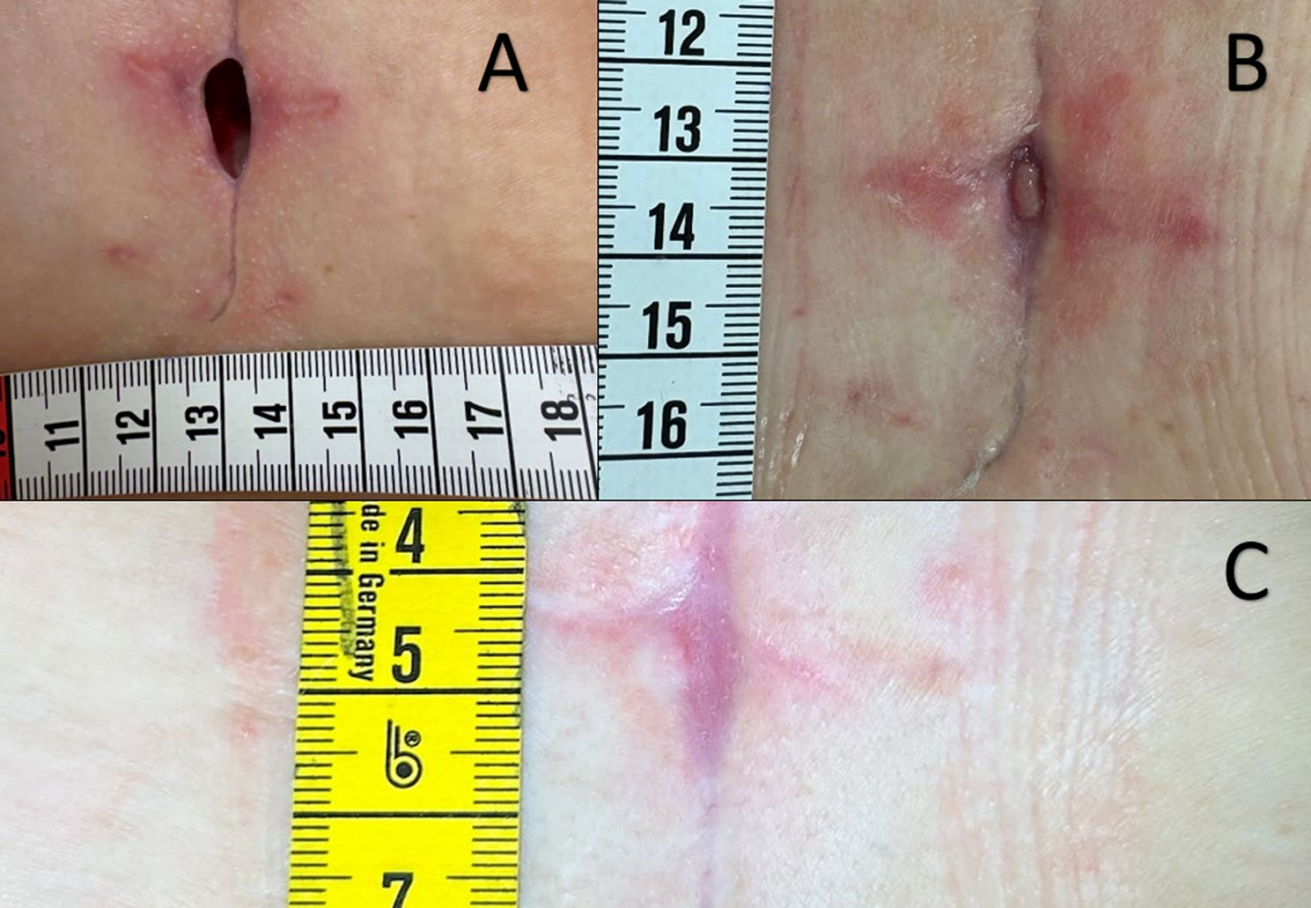
**A** Wound appearance after initiation of local taurolidine 2% irrigation (day 8): the wound bed is well vascularized, fibrin deposits are markedly reduced, and progressive granulation and epithelialization are evident. **B** Complete wound healing by secondary intention 33 days after initiation of local taurolidine 2% solution irrigation therapy, without the need for additional surgical intervention. **C** Final outcome demonstrating an excellent aesthetic result of the sternal scar after complete wound healing. Picture taken on day 46 after initiation of local taurolidine 2% solution irrigation

On the 23^rd^ postop day we decided to start with local treatment with Taurolidine 2%(TauroPharm GmbH, Bavaria, Germany). Taurolidine was applied every second day following local wound irrigation with normal saline. By the fifth day of treatment, wound cultures tested negative, and progressive healing was observed. The wound bed appeared well vascularized with granulation tissue, fibrin deposits were markedly reduced, and rapid epithelialization ensued, ultimately leading to healing by secondary intention (Fig. 2a).

The patient was discharged on postoperative day 29, at which point antibiotic therapy was transitioned from intravenous cefepime to oral trimethoprim/sulfamethoxazole (MIC ≤ 20) at a dose of 3 g/day for three weeks. As an outpatient, she received local applications of taurolidine 2% solution twice weekly for the first two weeks, followed by once-weekly treatments. Following initiation of local taurolidine 2% solution therapy, the wound bed appeared well vascularized with granulation tissue, fibrin deposits were markedly reduced, and rapid epithelialization was observed. By day 33 after starting local 2% taurolidine, the wound had fully healed by secondary intention (Fig. 2b). Finally, the scar exhibited an excellent aesthetic outcome(Fig. 2c).

## Discussion and conclusion

Sternal wound complications remain a major concern in cardiac surgery, as they are associated with substantial morbidity, mortality, and significant healthcare costs. DSWI occurs in approximately 0.8–6% of sternotomy procedures and prevention is therefore of paramount importance [3]. Established risk factors include obesity, breast hypertrophy, and diabetes, with *Staphylococcus aureus* being the most frequently isolated pathogen [2]. 

To better understand the pathophysiology of these wounds and to guide optimal management strategies, several classifications have been proposed:

A classification based on the timing and mechanism of the presentation includes three categories. *Type I wounds* occur within the first few days post-surgery and involve early wound separation with or without sternal instability; they are characterized by sero-sanguineous drainage in absence of cellulitis, costochondritis, or osteomyelitis. *Type II wounds* occur within the first few weeks and are characterized by purulent discharge, cellulitis, mediastinal suppuration, and positive cultures sometimes leading to fulminant mediastinitis. *Type III wounds* occur months to years after surgery and are distinguished by the presence of chronic draining sinus tracts, localized cellulitis, osteomyelitis, or retained foreign bodies [4]. 

Recently an anatomical description of sternal wound, including depth and location, has been shown to be more practical and useful. DSWI can involve skin, subcutaneous tissue and eventually the bone and have been classified as: *sternal instability without infection*, *deep sternal wound infection without sternal instability* and *deep wound infection with sternal instability* [5]. 

Our patient presented with a deep sternal wound infection in the absence of sternal instability, a condition frequently observed in individuals who, despite preserved sternal integrity, experience significant tension on the overlying skin and soft tissues. This scenario is commonly encountered in patients with obesity or in women with breast hypertrophy, both of which were present in our case.

Although *Klebsiella aerogenes* is not typically classified among the most aggressive pathogens in DSWI compared with *Staphylococcus aureus* or MRSA, the persistent culture positivity despite appropriate systemic antibiotics and repeated surgical wound cleansing suggested impaired local bacterial clearance. In this context, taurolidine was considered due to its broad-spectrum activity, anti-biofilm properties, and reported efficacy in cases where conventional therapy shows limited success. Moreover, the failure to achieve local sterilization prompted consideration of alternatives to VAC therapy, which we sought to avoid as the wound bed demonstrated early granulation potential and there was no sternal instability or mediastinitis.

There is a lack of evidence-based surgical consensus for the appropriate management strategy, including type of closure, choice of sternal coverage post sternotomy, and when to use reconstructive flaps.

In case of DSWI without sternal instability extensive debridement and aggressive antibiotic treatment are suggested [2, 3, 5]. VAC therapy may be beneficial until the wound is adequately debrided and granulation tissue has formed, while systemic intravenous antibiotic administration should be continued for at least six weeks. In cases involving the sternum or when mediastinitis is present, bone resection is often required, typically followed by bilateral pectoral muscle advancement flaps [2, 3, 5]. For accurate diagnosis, laboratory analyses are essential; [6] however, CT imaging is mandatory to rule out concomitant mediastinitis, which is frequently indicated by the presence of free gas, pleural effusions, or enlarged brachiocephalic lymph nodes.

In cases of refractory local infection, biofilm formation and persistent low-grade bacterial colonization may impede wound healing even in the absence of systemic signs. Taurolidine has demonstrated broad-spectrum antimicrobial activity, biofilm inhibition, and anti-inflammatory effects through cytokine suppression (TNF-α, IL-1, IL-6). These properties support its utility when traditional wound care and systemic antibiotics fail to fully eradicate bacterial presence. In our patient, taurolidine was initiated after repeated positive cultures and worsening wound appearance despite targeted intravenous antibiotics and meticulous wound care, serving as a targeted adjunct to infection control and wound healing optimization.

The decision to introduce taurolidine was based on persistent bacterial detection and progressive local deterioration, despite appropriate systemic therapy and local antiseptic protocols. Our aim was to control infection while preserving tissue and avoiding escalation to VAC therapy or surgical muscle flaps, which would have impeded outpatient management of our patient.

Taurolidine is a derivative of the amino acid taurine and exerts broad-spectrum antimicrobial activity. Its mechanism of action involves the release of N-methylol groups, which interact with microbial cell wall structures, leading to disruption of cell integrity and inhibition of biofilm formation. Taurolidine has demonstrated efficacy against a wide range of gram-positive and gram-negative bacteria, as well as activity against certain fungi and viruses [7]. Taurolidine is also effective in preventing biofilm formation [8]. In addition to its antimicrobial activity, it exhibits anti-inflammatory properties through the inhibition of proinflammatory cytokines such as TNF-α, IL-1, and IL-6. Taurine, its parent compound, has been associated with wound-healing effects [9]. When applied topically for irrigation, taurolidine is generally well tolerated and not associated with significant adverse effects, even after intravenous administration.

In the present case, renal and hepatic function were monitored weekly during hospitalization and every other week thereafter, with no alterations in creatinine, eGFR, or liver enzymes. Taurolidine 2% solution was applied at a volume of approximately 10 mL every 48 h during hospitalization. After hospital discharge the therapy was continued twice-weekly for two weeks and weekly applications, until complete closure. Follow-up was uneventful.

Taurolidine has proven to be effective in preventing infections associated with central venous access devices, by interfering with bacterial and fungal adhesion and growth, making it therefore an ideal tool for central venous access device blocking [10]. A distinguishing feature of taurolidine is its ability to exert its antimicrobial effects irreversibly, ensuring that bacteria and fungi do not develop resistance.

Taurolidine irrigation may be considered based on wound size, depth, tissue viability, and the presence of foreign material. In larger or deeper cavities, or when hardware is exposed, combination therapy with VAC and taurolidine, as described by Weichsel et al. for left-ventricular assist device driveline infections, can enhance infection control and promote granulation within a single intervention [11]. For long-term treatment aiming for healing by secondary intention, taurolidine may be applied twice daily for up to three days, then reduced to every other day as the wound improves or when thoracic drainage becomes clear to avoid potential overtreatment, with volumes up to 250 mL per application. In our case, the defect was small-to-moderate in depth, without sternal instability or foreign bodies, and demonstrated early granulation potential; only 10 mL 2% solution per application was required, making taurolidine monotherapy sufficient. These observations support a tailored approach in which taurolidine may be used either as a primary therapy or as an adjunct depending on wound characteristics and tissue involvement.

Local taurolidine irrigation significantly reduces the occurrence of minor infections in ankle fracture surgeries and has proved to be effective in treating deep pressure ulcers, which are a significant healthcare challenge often not responding to conventional therapies [12]. It has also been used to treat patients with cardiac device pocket infection including hardware protrusion, however in larger quantities (200–300 ml 2% solution per revision) [13]. It is employed to prevent infection form active cardiac implants as well, with quantities rarely exceeding 100mL 2% solution per application [14, 15]. 

Our case highlights the potential of taurolidine as an antiseptic, antimicrobial, and wound-healing agent in the management of DSWI refractory to conventional treatment. When used as a local irrigant, taurolidine led to complete wound healing within one month, thereby avoiding further surgical intervention and the need for advanced therapies such as negative pressure assisted closure. These findings suggest that taurolidine may represent a valuable adjunct in the treatment of complex wound infections. However, randomized controlled trials on a larger scale are warranted to establish its efficacy and define its role in this clinical context.

Based on this experience, taurolidine may be considered in selected cases of DSWI without sternal instability, shallow to moderate wound depth, and persistent colonization despite appropriate systemic therapy. Future studies are necessary to clarify its optimal dosing schedule, indications, and its role alongside or instead of VAC therapy in selected patients.
